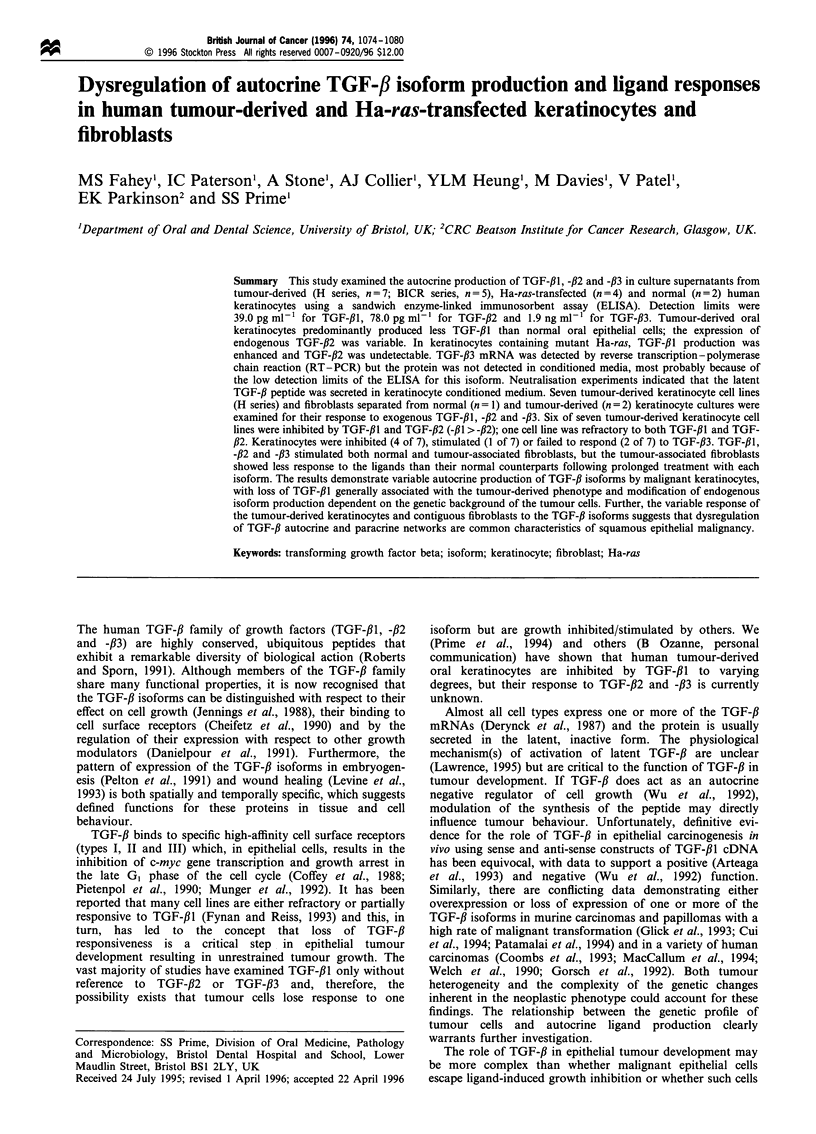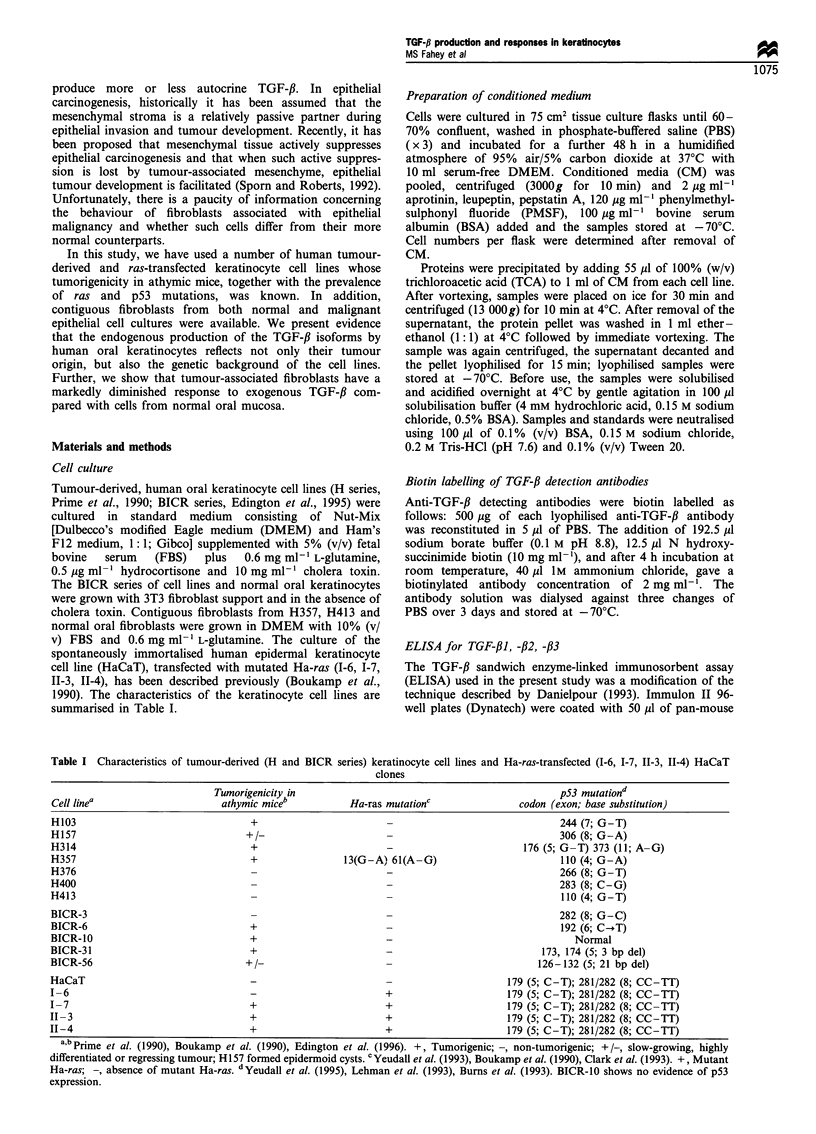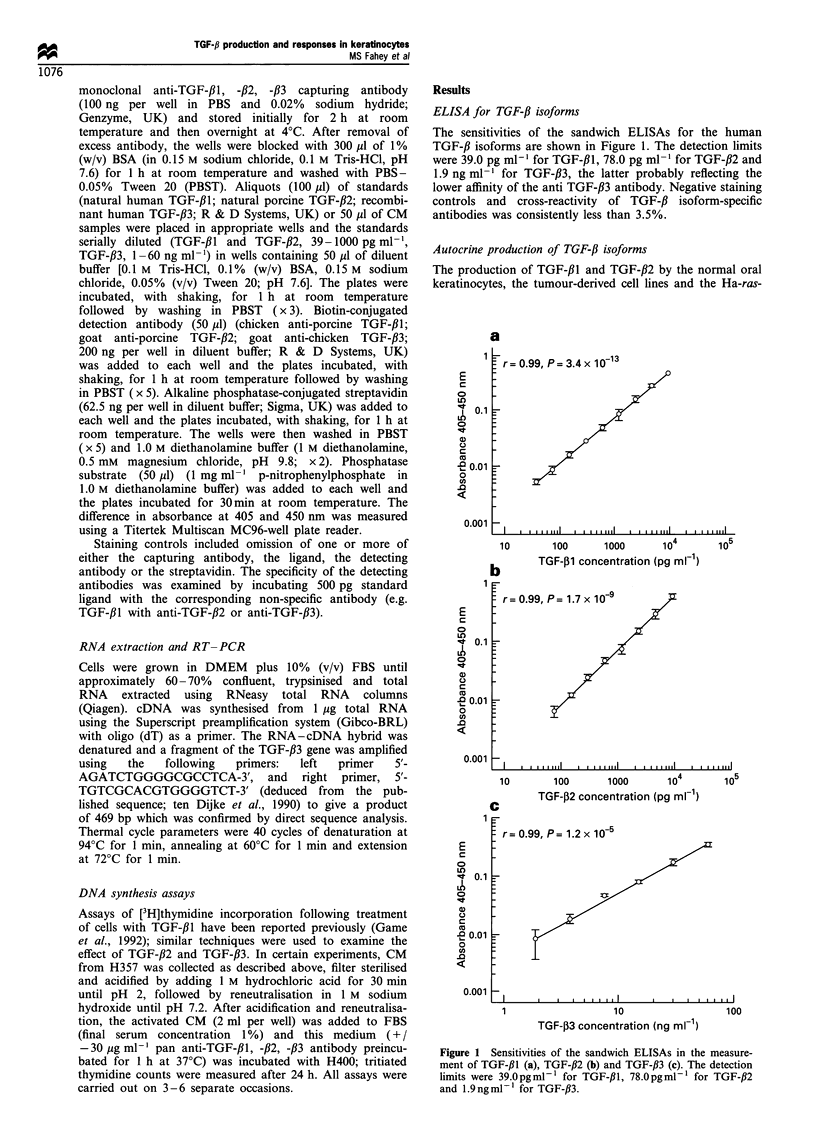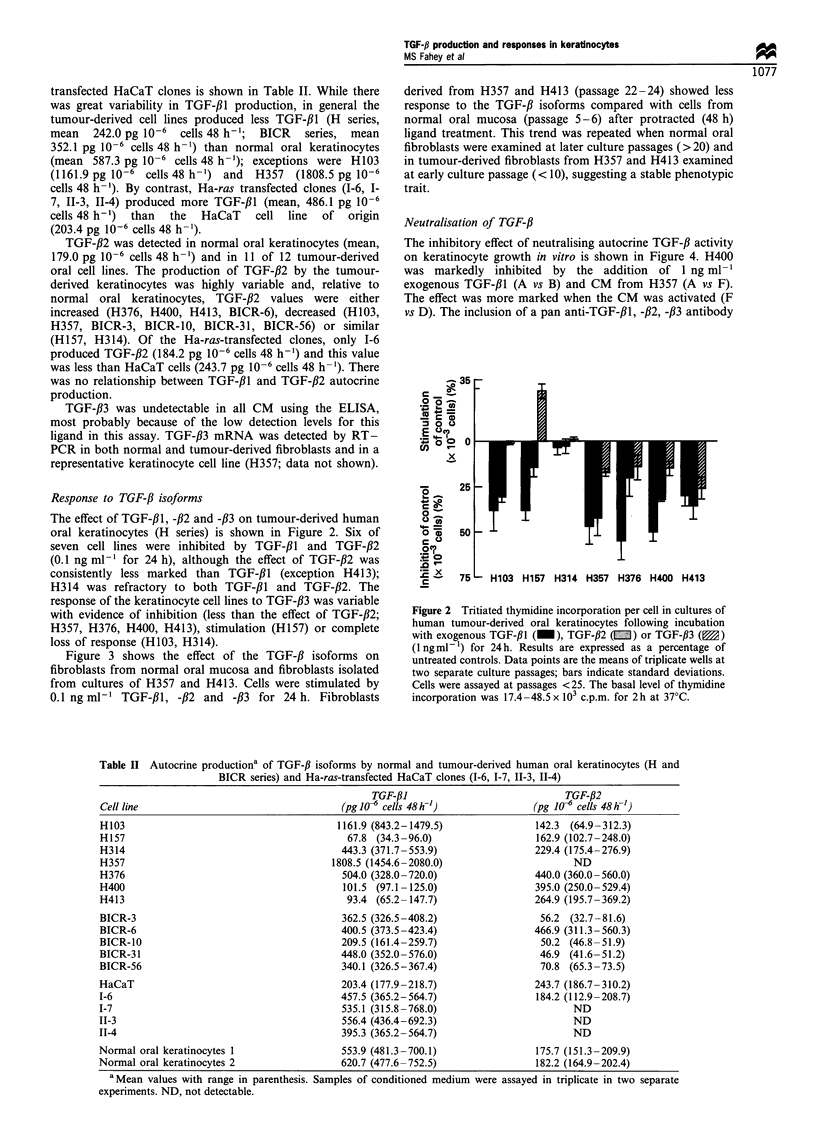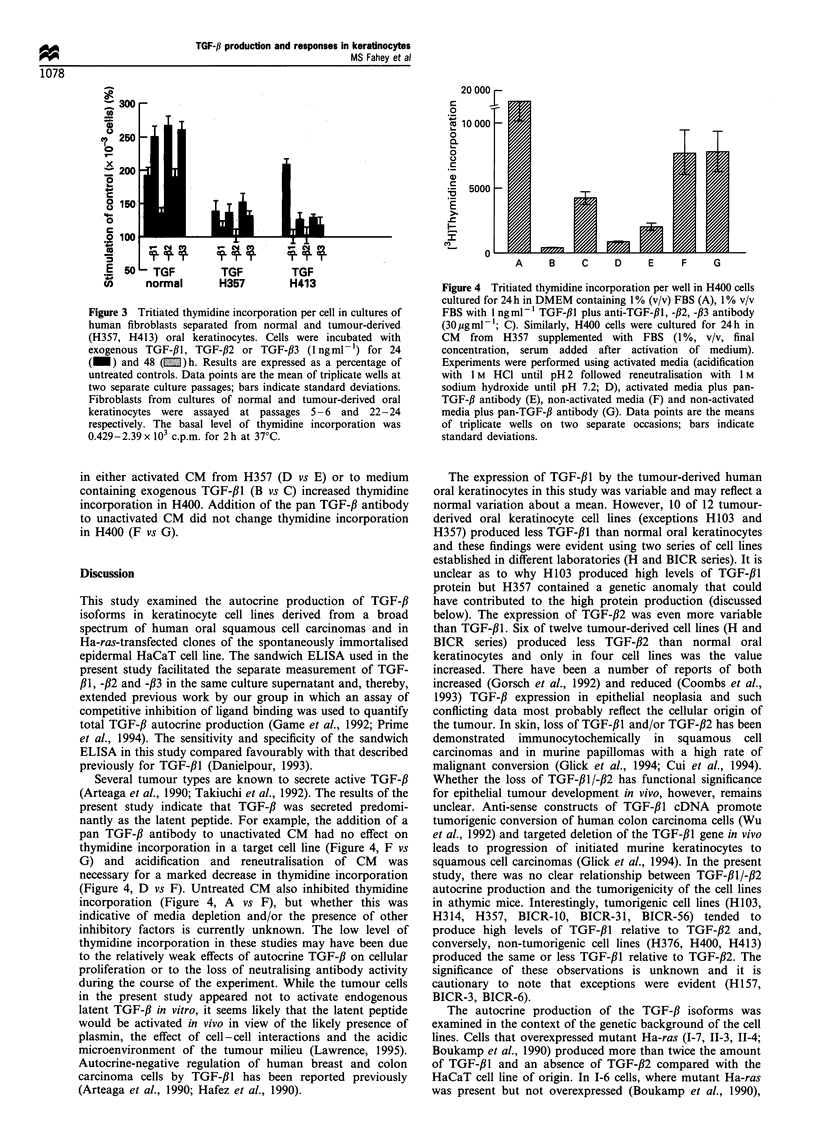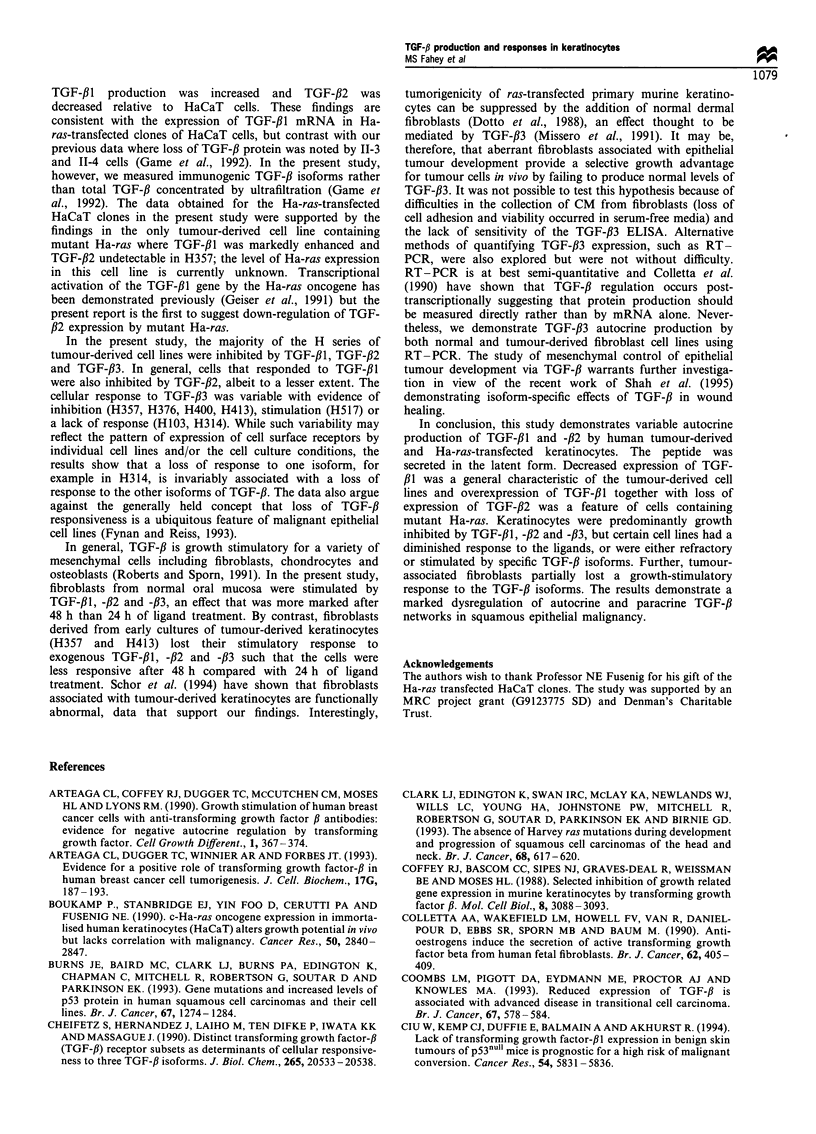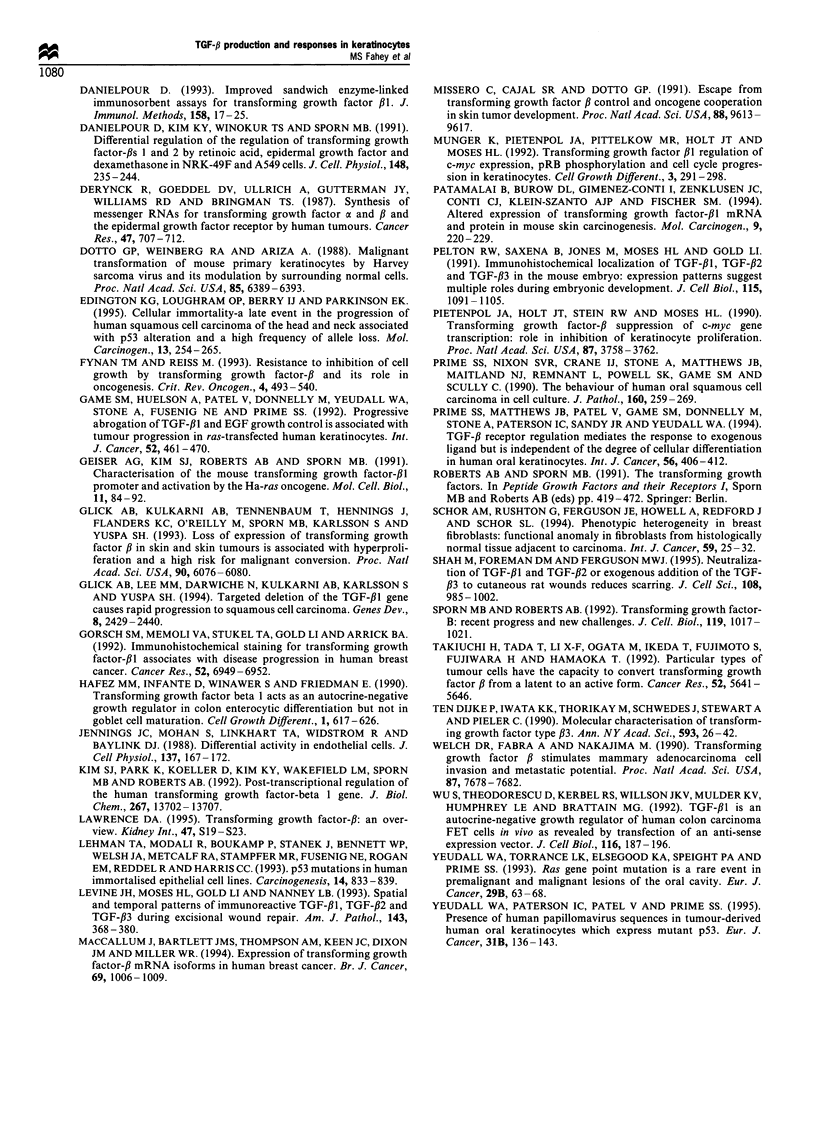# Dysregulation of autocrine TGF-beta isoform production and ligand responses in human tumour-derived and Ha-ras-transfected keratinocytes and fibroblasts.

**DOI:** 10.1038/bjc.1996.492

**Published:** 1996-10

**Authors:** M. S. Fahey, I. C. Paterson, A. Stone, A. J. Collier, Y. L. Heung, M. Davies, V. Patel, E. K. Parkinson, S. S. Prime

**Affiliations:** Department of Oral and Dental Science, University of Bristol, UK.

## Abstract

This study examined the autocrine production of TGF-beta 1, -beta 2 and -beta 3 in culture supernatants from tumour-derived (H series, n = 7; BICR series, n = 5), Ha-ras-transfected (n = 4) and normal (n = 2) human keratinocytes using a sandwich enzyme-linked immunosorbent assay (ELISA). Detection limits were 39.0 pg ml-1 for TGF-beta 1, 78.0 pg ml-1 for TGF-beta 2 and 1.9 ng ml-1 for TGF-beta 3. Tumour-derived oral keratinocytes predominantly produced less TGF-beta 1 than normal oral epithelial cells; the expression of endogenous TGF-beta 2 was variable. In keratinocytes containing mutant Ha-ras, TGF-beta 1 production was enhanced and TGF-beta 2 was undetectable. TGF-beta 3 mRNA was detected by reverse transcription-polymerase chain reaction (RT-PCR) but the protein was not detected in conditioned media, most probably because of the low detection limits of the ELISA for this isoform. Neutralisation experiments indicated that the latent TGF-beta peptide was secreted in keratinocyte conditioned medium. Seven tumour-derived keratinocyte cell lines (H series) and fibroblasts separated from normal (n = 1) and tumour-derived (n = 2) keratinocyte cultures were examined for their response to exogenous TGF-beta 1, -beta 2 and -beta 3. Six of seven tumour-derived keratinocyte cell lines were inhibited by TGF-beta 1 and TGF-beta 2 (-beta 1 > -beta 2); one cell line was refractory to both TGF-beta 1 and TGF-beta 2. Keratinocytes were inhibited (4 of 7), stimulated (1 of 7) or failed to respond (2 of 7) to TGF-beta 3, TGF-beta 1, -beta 2 and -beta 3 stimulated both normal and tumour-associated fibroblasts, but the tumour-associated fibroblasts showed less response to the ligands than their normal counterparts following prolonged treatment with each isoform. The results demonstrate variable autocrine production of TGF-beta isoforms by malignant keratinocytes, with loss of TGF-beta 1 generally associated with the tumour-derived phenotype and modification of endogenous isoform production dependent on the genetic background of the tumour cells. Further, the variable response of the tumour-derived keratinocytes and contiguous fibroblasts to the TGF-beta isoforms suggests that dysregulation of TGF-beta autocrine and paracrine networks are common characteristics of squamous epithelial malignancy.


British Journal of Cancer (1996) 74, 1074-1080
© 1996 Stockton Press All rights reserved 0007-0920/96 $12.00

### Dysregulation of autocrine TGF-$\beta$ isoform production and ligand responses in human tumour-derived and Ha-*ras*-transfected keratinocytes and fibroblasts

MS Fahey[1], IC Paterson[1], A Stone[1], AJ Collier[1], YLM Heung[1], M Davies[1], V Patel[1], EK Parkinson[2] and SS Prime[1]

[1]*Department of Oral and Dental Science, University of Bristol, UK;* [2]*CRC Beatson Institute for Cancer Research, Glasgow, UK.*

**Summary** This study examined the autocrine production of TGF-$\beta$1, -$\beta$2 and -$\beta$3 in culture supernatants from tumour-derived (H series, $n=7$; BICR series, $n=5$), Ha-*ras*-transfected ($n=4$) and normal ($n=2$) human keratinocytes using a sandwich enzyme-linked immunosorbent assay (ELISA). Detection limits were 39.0 pg ml$^{-1}$ for TGF-$\beta$1, 78.0 pg ml$^{-1}$ for TGF-$\beta$2 and 1.9 ng ml$^{-1}$ for TGF-$\beta$3. Tumour-derived oral keratinocytes predominantly produced less TGF-$\beta$1 than normal oral epithelial cells; the expression of endogenous TGF-$\beta$2 was variable. In keratinocytes containing mutant Ha-*ras*, TGF-$\beta$1 production was enhanced and TGF-$\beta$2 was undetectable. TGF-$\beta$3 mRNA was detected by reverse transcription-polymerase chain reaction (RT-PCR) but the protein was not detected in conditioned media, most probably because of the low detection limits of the ELISA for this isoform. Neutralisation experiments indicated that the latent TGF-$\beta$ peptide was secreted in keratinocyte conditioned medium. Seven tumour-derived keratinocyte cell lines (H series) and fibroblasts separated from normal ($n=1$) and tumour-derived ($n=2$) keratinocyte cultures were examined for their response to exogenous TGF-$\beta$1, -$\beta$2 and -$\beta$3. Six of seven tumour-derived keratinocyte cell lines were inhibited by TGF-$\beta$1 and TGF-$\beta$2 (-$\beta$1 > -$\beta$2); one cell line was refractory to both TGF-$\beta$1 and TGF-$\beta$2. Keratinocytes were inhibited (4 of 7), stimulated (1 of 7) or failed to respond (2 of 7) to TGF-$\beta$3. TGF-$\beta$1, -$\beta$2 and -$\beta$3 stimulated both normal and tumour-associated fibroblasts, but the tumour-associated fibroblasts showed less response to the ligands than their normal counterparts following prolonged treatment with each isoform. The results demonstrate variable autocrine production of TGF-$\beta$ isoforms by malignant keratinocytes, with loss of TGF-$\beta$1 generally associated with the tumour-derived phenotype and modification of endogenous isoform production dependent on the genetic background of the tumour cells. Further, the variable response of the tumour-derived keratinocytes and contiguous fibroblasts to the TGF-$\beta$ isoforms suggests that dysregulation of TGF-$\beta$ autocrine and paracrine networks are common characteristics of squamous epithelial malignancy.

**Keywords:** transforming growth factor beta; isoform; keratinocyte; fibroblast; Ha-*ras*

The human TGF-$\beta$ family of growth factors (TGF-$\beta$1, -$\beta$2 and -$\beta$3) are highly conserved, ubiquitous peptides that exhibit a remarkable diversity of biological action (Roberts and Sporn, 1991). Although members of the TGF-$\beta$ family share many functional properties, it is now recognised that the TGF-$\beta$ isoforms can be distinguished with respect to their effect on cell growth (Jennings *et al.*, 1988), their binding to cell surface receptors (Cheifetz *et al.*, 1990) and by the regulation of their expression with respect to other growth modulators (Danielpour *et al.*, 1991). Furthermore, the pattern of expression of the TGF-$\beta$ isoforms in embryogenesis (Pelton *et al.*, 1991) and wound healing (Levine *et al.*, 1993) is both spatially and temporally specific, which suggests defined functions for these proteins in tissue and cell behaviour.

TGF-$\beta$ binds to specific high-affinity cell surface receptors (types I, II and III) which, in epithelial cells, results in the inhibition of c-*myc* gene transcription and growth arrest in the late $G_1$ phase of the cell cycle (Coffey *et al.*, 1988; Pietenpol *et al.*, 1990; Munger *et al.*, 1992). It has been reported that many cell lines are either refractory or partially responsive to TGF-$\beta$1 (Fynan and Reiss, 1993) and this, in turn, has led to the concept that loss of TGF-$\beta$ responsiveness is a critical step in epithelial tumour development resulting in unrestrained tumour growth. The vast majority of studies have examined TGF-$\beta$1 only without reference to TGF-$\beta$2 or TGF-$\beta$3 and, therefore, the possibility exists that tumour cells lose response to one isoform but are growth inhibited/stimulated by others. We (Prime *et al.*, 1994) and others (B Ozanne, personal communication) have shown that human tumour-derived oral keratinocytes are inhibited by TGF-$\beta$1 to varying degrees, but their response to TGF-$\beta$2 and -$\beta$3 is currently unknown.

Almost all cell types express one or more of the TGF-$\beta$ mRNAs (Derynck *et al.*, 1987) and the protein is usually secreted in the latent, inactive form. The physiological mechanism(s) of activation of latent TGF-$\beta$ are unclear (Lawrence, 1995) but are critical to the function of TGF-$\beta$ in tumour development. If TGF-$\beta$ does act as an autocrine negative regulator of cell growth (Wu *et al.*, 1992), modulation of the synthesis of the peptide may directly influence tumour behaviour. Unfortunately, definitive evidence for the role of TGF-$\beta$ in epithelial carcinogenesis *in vivo* using sense and anti-sense constructs of TGF-$\beta$1 cDNA has been equivocal, with data to support a positive (Arteaga *et al.*, 1993) and negative (Wu *et al.*, 1992) function. Similarly, there are conflicting data demonstrating either overexpression or loss of expression of one or more of the TGF-$\beta$ isoforms in murine carcinomas and papillomas with a high rate of malignant transformation (Glick *et al.*, 1993; Cui *et al.*, 1994; Patamalai *et al.*, 1994) and in a variety of human carcinomas (Coombs *et al.*, 1993; MacCallum *et al.*, 1994; Welch *et al.*, 1990; Gorsch *et al.*, 1992). Both tumour heterogeneity and the complexity of the genetic changes inherent in the neoplastic phenotype could account for these findings. The relationship between the genetic profile of tumour cells and autocrine ligand production clearly warrants further investigation.

The role of TGF-$\beta$ in epithelial tumour development may be more complex than whether malignant epithelial cells escape ligand-induced growth inhibition or whether such cells

Correspondence: SS Prime, Division of Oral Medicine, Pathology and Microbiology, Bristol Dental Hospital and School, Lower Maudlin Street, Bristol BS1 2LY, UK
Received 24 July 1995; revised 1 April 1996; accepted 22 April 1996

produce more or less autocrine TGF-β. In epithelial carcinogenesis, historically it has been assumed that the mesenchymal stroma is a relatively passive partner during epithelial invasion and tumour development. Recently, it has been proposed that mesenchymal tissue actively suppresses epithelial carcinogenesis and that when such active suppression is lost by tumour-associated mesenchyme, epithelial tumour development is facilitated (Sporn and Roberts, 1992). Unfortunately, there is a paucity of information concerning the behaviour of fibroblasts associated with epithelial malignancy and whether such cells differ from their more normal counterparts.

In this study, we have used a number of human tumour-derived and *ras*-transfected keratinocyte cell lines whose tumorigenicity in athymic mice, together with the prevalence of *ras* and p53 mutations, was known. In addition, contiguous fibroblasts from both normal and malignant epithelial cell cultures were available. We present evidence that the endogenous production of the TGF-β isoforms by human oral keratinocytes reflects not only their tumour origin, but also the genetic background of the cell lines. Further, we show that tumour-associated fibroblasts have a markedly diminished response to exogenous TGF-β compared with cells from normal oral mucosa.

### Materials and methods

### *Cell culture*

Tumour-derived, human oral keratinocyte cell lines (H series, Prime *et al.*, 1990; BICR series, Edington *et al.*, 1995) were cultured in standard medium consisting of Nut-Mix [Dulbecco's modified Eagle medium (DMEM) and Ham's F12 medium, 1:1; Gibco] supplemented with 5% (v/v) fetal bovine serum (FBS) plus 0.6 mg ml$^{-1}$ L-glutamine, 0.5 μg ml$^{-1}$ hydrocortisone and 10 mg ml$^{-1}$ cholera toxin. The BICR series of cell lines and normal oral keratinocytes were grown with 3T3 fibroblast support and in the absence of cholera toxin. Contiguous fibroblasts from H357, H413 and normal oral fibroblasts were grown in DMEM with 10% (v/v) FBS and 0.6 mg ml$^{-1}$ L-glutamine. The culture of the spontaneously immortalised human epidermal keratinocyte cell line (HaCaT), transfected with mutated Ha-*ras* (I-6, I-7, II-3, II-4), has been described previously (Boukamp *et al.*, 1990). The characteristics of the keratinocyte cell lines are summarised in Table I.

### *Preparation of conditioned medium*

Cells were cultured in 75 cm$^2$ tissue culture flasks until 60–70% confluent, washed in phosphate-buffered saline (PBS) (×3) and incubated for a further 48 h in a humidified atmosphere of 95% air/5% carbon dioxide at 37°C with 10 ml serum-free DMEM. Conditioned media (CM) was pooled, centrifuged (3000*g* for 10 min) and 2 μg ml$^{-1}$ aprotinin, leupeptin, pepstatin A, 120 μg ml$^{-1}$ phenylmethylsulphonyl fluoride (PMSF), 100 μg ml$^{-1}$ bovine serum albumin (BSA) added and the samples stored at −70°C. Cell numbers per flask were determined after removal of CM.

Proteins were precipitated by adding 55 μl of 100% (w/v) trichloroacetic acid (TCA) to 1 ml of CM from each cell line. After vortexing, samples were placed on ice for 30 min and centrifuged (13 000*g*) for 10 min at 4°C. After removal of the supernatant, the protein pellet was washed in 1 ml ether–ethanol (1:1) at 4°C followed by immediate vortexing. The sample was again centrifuged, the supernatant decanted and the pellet lyophilised for 15 min; lyophilised samples were stored at −70°C. Before use, the samples were solubilised and acidified overnight at 4°C by gentle agitation in 100 μl solubilisation buffer (4 mM hydrochloric acid, 0.15 M sodium chloride, 0.5% BSA). Samples and standards were neutralised using 100 μl of 0.1% (v/v) BSA, 0.15 M sodium chloride, 0.2 M Tris-HCl (pH 7.6) and 0.1% (v/v) Tween 20.

### *Biotin labelling of TGF-β detection antibodies*

Anti-TGF-β detecting antibodies were biotin labelled as follows: 500 μg of each lyophilised anti-TGF-β antibody was reconstituted in 5 μl of PBS. The addition of 192.5 μl sodium borate buffer (0.1 M pH 8.8), 12.5 μl N hydroxy-succinimide biotin (10 mg ml$^{-1}$), and after 4 h incubation at room temperature, 40 μl 1M ammonium chloride, gave a biotinylated antibody concentration of 2 mg ml$^{-1}$. The antibody solution was dialysed against three changes of PBS over 3 days and stored at −70°C.

### *ELISA for TGF-β1, -β2, -β3*

The TGF-β sandwich enzyme-linked immunosorbent assay (ELISA) used in the present study was a modification of the technique described by Danielpour (1993). Immulon II 96-well plates (Dynatech) were coated with 50 μl of pan-mouse

**Table I** Characteristics of tumour-derived (H and BICR series) keratinocyte cell lines and Ha-*ras*-transfected (I-6, I-7, II-3, II-4) HaCaT clones

| *Cell line*[a] | *Tumorigenicity in athymic mice*[b] | *Ha*-ras *mutation*[c] | *p53 mutation*[d] *codon (exon; base substitution)* |
|---|---|---|---|
| H103 | + | – | 244 (7; G–T) |
| H157 | +/– | – | 306 (8; G–A) |
| H314 | + | – | 176 (5; G–T) 373 (11; A–G) |
| H357 | + | 13(G–A) 61(A–G) | 110 (4; G–A) |
| H376 | – | – | 266 (8; G–T) |
| H400 | – | – | 283 (8; C–G) |
| H413 | – | – | 110 (4; G–T) |
| BICR-3 | – | – | 282 (8; G–C) |
| BICR-6 | + | – | 192 (6; C→T) |
| BICR-10 | + | – | Normal |
| BICR-31 | + | – | 173, 174 (5; 3 bp del) |
| BICR-56 | +/– | – | 126–132 (5; 21 bp del) |
| HaCaT | – | – | 179 (5; C–T); 281/282 (8; CC–TT) |
| I–6 | – | + | 179 (5; C–T); 281/282 (8; CC–TT) |
| I–7 | + | + | 179 (5; C–T); 281/282 (8; CC–TT) |
| II–3 | + | + | 179 (5; C–T); 281/282 (8; CC–TT) |
| II–4 | + | + | 179 (5; C–T); 281/282 (8; CC–TT) |

[a,b] Prime *et al.* (1990), Boukamp *et al.* (1990), Edington *et al.* (1996). +, Tumorigenic; –, non-tumorigenic; +/–, slow-growing, highly differentiated or regressing tumour; H157 formed epidermoid cysts. [c] Yeudall *et al.* (1993), Boukamp *et al.* (1990), Clark *et al.* (1993). +, Mutant Ha-*ras*; –, absence of mutant Ha-*ras*. [d] Yeudall *et al.* (1995), Lehman *et al.* (1993), Burns *et al.* (1993). BICR-10 shows no evidence of p53 expression.

monoclonal anti-TGF-$\beta$1, -$\beta$2, -$\beta$3 capturing antibody (100 ng per well in PBS and 0.02% sodium hydride; Genzyme, UK) and stored initially for 2 h at room temperature and then overnight at 4°C. After removal of excess antibody, the wells were blocked with 300 $\mu$l of 1% (w/v) BSA (in 0.15 M sodium chloride, 0.1 M Tris-HCl, pH 7.6) for 1 h at room temperature and washed with PBS–0.05% Tween 20 (PBST). Aliquots (100 $\mu$l) of standards (natural human TGF-$\beta$1; natural porcine TGF-$\beta$2; recombinant human TGF-$\beta$3; R & D Systems, UK) or 50 $\mu$l of CM samples were placed in appropriate wells and the standards serially diluted (TGF-$\beta$1 and TGF-$\beta$2, 39–1000 pg ml$^{-1}$, TGF-$\beta$3, 1–60 ng ml$^{-1}$) in wells containing 50 $\mu$l of diluent buffer [0.1 M Tris-HCl, 0.1% (w/v) BSA, 0.15 M sodium chloride, 0.05% (v/v) Tween 20; pH 7.6]. The plates were incubated, with shaking, for 1 h at room temperature followed by washing in PBST ($\times$3). Biotin-conjugated detection antibody (50 $\mu$l) (chicken anti-porcine TGF-$\beta$1; goat anti-porcine TGF-$\beta$2; goat anti-chicken TGF-$\beta$3; 200 ng per well in diluent buffer; R & D Systems, UK) was added to each well and the plates incubated, with shaking, for 1 h at room temperature followed by washing in PBST ($\times$5). Alkaline phosphatase-conjugated streptavidin (62.5 ng per well in diluent buffer; Sigma, UK) was added to each well and the plates incubated, with shaking, for 1 h at room temperature. The wells were then washed in PBST ($\times$5) and 1.0 M diethanolamine buffer (1 M diethanolamine, 0.5 mM magnesium chloride, pH 9.8; $\times$2). Phosphatase substrate (50 $\mu$l) (1 mg ml$^{-1}$ p-nitrophenylphosphate in 1.0 M diethanolamine buffer) was added to each well and the plates incubated for 30 min at room temperature. The difference in absorbance at 405 and 450 nm was measured using a Titertek Multiscan MC96-well plate reader.

Staining controls included omission of one or more of either the capturing antibody, the ligand, the detecting antibody or the streptavidin. The specificity of the detecting antibodies was examined by incubating 500 pg standard ligand with the corresponding non-specific antibody (e.g. TGF-$\beta$1 with anti-TGF-$\beta$2 or anti-TGF-$\beta$3).

### *RNA extraction and RT–PCR*

Cells were grown in DMEM plus 10% (v/v) FBS until approximately 60–70% confluent, trypsinised and total RNA extracted using RNeasy total RNA columns (Qiagen). cDNA was synthesised from 1 $\mu$g total RNA using the Superscript preamplification system (Gibco-BRL) with oligo (dT) as a primer. The RNA–cDNA hybrid was denatured and a fragment of the TGF-$\beta$3 gene was amplified using the following primers: left primer 5′-AGATCTGGGGCGCCTCA-3′, and right primer, 5′-TGTCGCACGTGGGGTCT-3′ (deduced from the published sequence; ten Dijke *et al.*, 1990) to give a product of 469 bp which was confirmed by direct sequence analysis. Thermal cycle parameters were 40 cycles of denaturation at 94°C for 1 min, annealing at 60°C for 1 min and extension at 72°C for 1 min.

### *DNA synthesis assays*

Assays of [$^3$H]thymidine incorporation following treatment of cells with TGF-$\beta$1 have been reported previously (Game *et al.*, 1992); similar techniques were used to examine the effect of TGF-$\beta$2 and TGF-$\beta$3. In certain experiments, CM from H357 was collected as described above, filter sterilised and acidified by adding 1 M hydrochloric acid for 30 min until pH 2, followed by reneutralisation in 1 M sodium hydroxide until pH 7.2. After acidification and reneutralisation, the activated CM (2 ml per well) was added to FBS (final serum concentration 1%) and this medium (+/−30 $\mu$g ml$^{-1}$ pan anti-TGF-$\beta$1, -$\beta$2, -$\beta$3 antibody preincubated for 1 h at 37°C) was incubated with H400; tritiated thymidine counts were measured after 24 h. All assays were carried out on 3–6 separate occasions.

### Results

### *ELISA for TGF-$\beta$ isoforms*

The sensitivities of the sandwich ELISAs for the human TGF-$\beta$ isoforms are shown in Figure 1. The detection limits were 39.0 pg ml$^{-1}$ for TGF-$\beta$1, 78.0 pg ml$^{-1}$ for TGF-$\beta$2 and 1.9 ng ml$^{-1}$ for TGF-$\beta$3, the latter probably reflecting the lower affinity of the anti TGF-$\beta$3 antibody. Negative staining controls and cross-reactivity of TGF-$\beta$ isoform-specific antibodies was consistently less than 3.5%.

### *Autocrine production of TGF-$\beta$ isoforms*

The production of TGF-$\beta$1 and TGF-$\beta$2 by the normal oral keratinocytes, the tumour-derived cell lines and the Ha-*ras*-

**Figure 1** Sensitivities of the sandwich ELISAs in the measurement of TGF-$\beta$1 (**a**), TGF-$\beta$2 (**b**) and TGF-$\beta$3 (**c**). The detection limits were 39.0 pg ml$^{-1}$ for TGF-$\beta$1, 78.0 pg ml$^{-1}$ for TGF-$\beta$2 and 1.9 ng ml$^{-1}$ for TGF-$\beta$3.

transfected HaCaT clones is shown in Table II. While there was great variability in TGF-β1 production, in general the tumour-derived cell lines produced less TGF-β1 (H series, mean 242.0 pg $10^{-6}$ cells 48 $h^{-1}$; BICR series, mean 352.1 pg $10^{-6}$ cells 48 $h^{-1}$) than normal oral keratinocytes (mean 587.3 pg $10^{-6}$ cells 48 $h^{-1}$); exceptions were H103 (1161.9 pg $10^{-6}$ cells 48 $h^{-1}$) and H357 (1808.5 pg $10^{-6}$ cells 48 $h^{-1}$). By contrast, Ha-*ras* transfected clones (I-6, I-7, II-3, II-4) produced more TGF-β1 (mean, 486.1 pg $10^{-6}$ cells 48 $h^{-1}$) than the HaCaT cell line of origin (203.4 pg $10^{-6}$ cells 48 $h^{-1}$).

TGF-β2 was detected in normal oral keratinocytes (mean, 179.0 pg $10^{-6}$ cells 48 $h^{-1}$) and in 11 of 12 tumour-derived oral cell lines. The production of TGF-β2 by the tumour-derived keratinocytes was highly variable and, relative to normal oral keratinocytes, TGF-β2 values were either increased (H376, H400, H413, BICR-6), decreased (H103, H357, BICR-3, BICR-10, BICR-31, BICR-56) or similar (H157, H314). Of the Ha-*ras*-transfected clones, only I-6 produced TGF-β2 (184.2 pg $10^{-6}$ cells 48 $h^{-1}$) and this value was less than HaCaT cells (243.7 pg $10^{-6}$ cells 48 $h^{-1}$). There was no relationship between TGF-β1 and TGF-β2 autocrine production.

TGF-β3 was undetectable in all CM using the ELISA, most probably because of the low detection levels for this ligand in this assay. TGF-β3 mRNA was detected by RT-PCR in both normal and tumour-derived fibroblasts and in a representative keratinocyte cell line (H357; data not shown).

*Response to TGF-β isoforms*

The effect of TGF-β1, -β2 and -β3 on tumour-derived human oral keratinocytes (H series) is shown in Figure 2. Six of seven cell lines were inhibited by TGF-β1 and TGF-β2 (0.1 ng $ml^{-1}$ for 24 h), although the effect of TGF-β2 was consistently less marked than TGF-β1 (exception H413); H314 was refractory to both TGF-β1 and TGF-β2. The response of the keratinocyte cell lines to TGF-β3 was variable with evidence of inhibition (less than the effect of TGF-β2; H357, H376, H400, H413), stimulation (H157) or complete loss of response (H103, H314).

Figure 3 shows the effect of the TGF-β isoforms on fibroblasts from normal oral mucosa and fibroblasts isolated from cultures of H357 and H413. Cells were stimulated by 0.1 ng $ml^{-1}$ TGF-β1, -β2 and -β3 for 24 h. Fibroblasts derived from H357 and H413 (passage 22–24) showed less response to the TGF-β isoforms compared with cells from normal oral mucosa (passage 5–6) after protracted (48 h) ligand treatment. This trend was repeated when normal oral fibroblasts were examined at later culture passages (>20) and in tumour-derived fibroblasts from H357 and H413 examined at early culture passage (<10), suggesting a stable phenotypic trait.

*Neutralisation of TGF-β*

The inhibitory effect of neutralising autocrine TGF-β activity on keratinocyte growth *in vitro* is shown in Figure 4. H400 was markedly inhibited by the addition of 1 ng $ml^{-1}$ exogenous TGF-β1 (A *vs* B) and CM from H357 (A *vs* F). The effect was more marked when the CM was activated (F *vs* D). The inclusion of a pan anti-TGF-β1, -β2, -β3 antibody

**Figure 2** Tritiated thymidine incorporation per cell in cultures of human tumour-derived oral keratinocytes following incubation with exogenous TGF-β1 (■), TGF-β2 (▨) or TGF-β3 (▨) (1 ng $ml^{-1}$) for 24 h. Results are expressed as a percentage of untreated controls. Data points are the means of triplicate wells at two separate culture passages; bars indicate standard deviations. Cells were assayed at passages <25. The basal level of thymidine incorporation was 17.4–48.5 x $10^3$ c.p.m. for 2 h at 37°C.

**Table II** Autocrine production[a] of TGF-β isoforms by normal and tumour-derived human oral keratinocytes (H and BICR series) and Ha-*ras*-transfected HaCaT clones (I-6, I-7, II-3, II-4)

| *Cell line* | *TGF-β1 (pg $10^{-6}$ cells 48 $h^{-1}$)* | *TGF-β2 (pg $10^{-6}$ cells 48 $h^{-1}$)* |
|---|---|---|
| H103 | 1161.9 (843.2–1479.5) | 142.3 (64.9–312.3) |
| H157 | 67.8 (34.3–96.0) | 162.9 (102.7–248.0) |
| H314 | 443.3 (371.7–553.9) | 229.4 (175.4–276.9) |
| H357 | 1808.5 (1454.6–2080.0) | ND |
| H376 | 504.0 (328.0–720.0) | 440.0 (360.0–560.0) |
| H400 | 101.5 (97.1–125.0) | 395.0 (250.0–529.4) |
| H413 | 93.4 (65.2–147.7) | 264.9 (195.7–369.2) |
| BICR-3 | 362.5 (326.5–408.2) | 56.2 (32.7–81.6) |
| BICR-6 | 400.5 (373.5–423.4) | 466.9 (311.3–560.3) |
| BICR-10 | 209.5 (161.4–259.7) | 50.2 (46.8–51.9) |
| BICR-31 | 448.0 (352.0–576.0) | 46.9 (41.6–51.2) |
| BICR-56 | 340.1 (326.5–367.4) | 70.8 (65.3–73.5) |
| HaCaT | 203.4 (177.9–218.7) | 243.7 (186.7–310.2) |
| I-6 | 457.5 (365.2–564.7) | 184.2 (112.9–208.7) |
| I-7 | 535.1 (315.8–768.0) | ND |
| II-3 | 556.4 (436.4–692.3) | ND |
| II-4 | 395.3 (365.2–564.7) | ND |
| Normal oral keratinocytes 1 | 553.9 (481.3–700.1) | 175.7 (151.3–209.9) |
| Normal oral keratinocytes 2 | 620.7 (477.6–752.5) | 182.2 (164.9–202.4) |

[a] Mean values with range in parenthesis. Samples of conditioned medium were assayed in triplicate in two separate experiments. ND, not detectable.

**Figure 3** Tritiated thymidine incorporation per cell in cultures of human fibroblasts separated from normal and tumour-derived (H357, H413) oral keratinocytes. Cells were incubated with exogenous TGF-β1, TGF-β2 or TGF-β3 (1 ng ml$^{-1}$) for 24 (■) and 48 (▒) h. Results are expressed as a percentage of untreated controls. Data points are the mean of triplicate wells at two separate culture passages; bars indicate standard deviations. Fibroblasts from cultures of normal and tumour-derived oral keratinocytes were assayed at passages 5–6 and 22–24 respectively. The basal level of thymidine incorporation was 0.429–2.39 × $10^3$ c.p.m. for 2 h at 37°C.

**Figure 4** Tritiated thymidine incorporation per well in H400 cells cultured for 24 h in DMEM containing 1% (v/v) FBS (A), 1% v/v FBS with 1 ng ml$^{-1}$ TGF-β1 plus anti-TGF-β1, -β2, -β3 antibody (30 μg ml$^{-1}$; C). Similarly, H400 cells were cultured for 24 h in CM from H357 supplemented with FBS (1%, v/v, final concentration, serum added after activation of medium). Experiments were performed using activated media (acidification with 1 M HCl until pH 2 followed reneutralisation with 1 M sodium hydroxide until pH 7.2; D), activated media plus pan-TGF-β antibody (E), non-activated media (F) and non-activated media plus pan-TGF-β antibody (G). Data points are the means of triplicate wells on two separate occasions; bars indicate standard deviations.

in either activated CM from H357 (D *vs* E) or to medium containing exogenous TGF-β1 (B *vs* C) increased thymidine incorporation in H400. Addition of the pan TGF-β antibody to unactivated CM did not change thymidine incorporation in H400 (F *vs* G).

### Discussion

This study examined the autocrine production of TGF-β isoforms in keratinocyte cell lines derived from a broad spectrum of human oral squamous cell carcinomas and in Ha-*ras*-transfected clones of the spontaneously immortalised epidermal HaCaT cell line. The sandwich ELISA used in the present study facilitated the separate measurement of TGF-β1, -β2 and -β3 in the same culture supernatant and, thereby, extended previous work by our group in which an assay of competitive inhibition of ligand binding was used to quantify total TGF-β autocrine production (Game *et al.*, 1992; Prime *et al.*, 1994). The sensitivity and specificity of the sandwich ELISA in this study compared favourably with that described previously for TGF-β1 (Danielpour, 1993).

Several tumour types are known to secrete active TGF-β (Arteaga *et al.*, 1990; Takiuchi *et al.*, 1992). The results of the present study indicate that TGF-β was secreted predominantly as the latent peptide. For example, the addition of a pan TGF-β antibody to unactivated CM had no effect on thymidine incorporation in a target cell line (Figure 4, F *vs* G) and acidification and reneutralisation of CM was necessary for a marked decrease in thymidine incorporation (Figure 4, D *vs* F). Untreated CM also inhibited thymidine incorporation (Figure 4, A *vs* F), but whether this was indicative of media depletion and/or the presence of other inhibitory factors is currently unknown. The low level of thymidine incorporation in these studies may have been due to the relatively weak effects of autocrine TGF-β on cellular proliferation or to the loss of neutralising antibody activity during the course of the experiment. While the tumour cells in the present study appeared not to activate endogenous latent TGF-β *in vitro*, it seems likely that the latent peptide would be activated *in vivo* in view of the likely presence of plasmin, the effect of cell–cell interactions and the acidic microenvironment of the tumour milieu (Lawrence, 1995). Autocrine-negative regulation of human breast and colon carcinoma cells by TGF-β1 has been reported previously (Arteaga *et al.*, 1990; Hafez *et al.*, 1990).

The expression of TGF-β1 by the tumour-derived human oral keratinocytes in this study was variable and may reflect a normal variation about a mean. However, 10 of 12 tumour-derived oral keratinocyte cell lines (exceptions H103 and H357) produced less TGF-β1 than normal oral keratinocytes and these findings were evident using two series of cell lines established in different laboratories (H and BICR series). It is unclear as to why H103 produced high levels of TGF-β1 protein but H357 contained a genetic anomaly that could have contributed to the high protein production (discussed below). The expression of TGF-β2 was even more variable than TGF-β1. Six of twelve tumour-derived cell lines (H and BICR series) produced less TGF-β2 than normal oral keratinocytes and only in four cell lines was the value increased. There have been a number of reports of both increased (Gorsch *et al.*, 1992) and reduced (Coombs *et al.*, 1993) TGF-β expression in epithelial neoplasia and such conflicting data most probably reflect the cellular origin of the tumour. In skin, loss of TGF-β1 and/or TGF-β2 has been demonstrated immunocytochemically in squamous cell carcinomas and in murine papillomas with a high rate of malignant conversion (Glick *et al.*, 1994; Cui *et al.*, 1994). Whether the loss of TGF-β1/-β2 has functional significance for epithelial tumour development *in vivo*, however, remains unclear. Anti-sense constructs of TGF-β1 cDNA promote tumorigenic conversion of human colon carcinoma cells (Wu *et al.*, 1992) and targeted deletion of the TGF-β1 gene *in vivo* leads to progression of initiated murine keratinocytes to squamous cell carcinomas (Glick *et al.*, 1994). In the present study, there was no clear relationship between TGF-β1/-β2 autocrine production and the tumorigenicity of the cell lines in athymic mice. Interestingly, tumorigenic cell lines (H103, H314, H357, BICR-10, BICR-31, BICR-56) tended to produce high levels of TGF-β1 relative to TGF-β2 and, conversely, non-tumorigenic cell lines (H376, H400, H413) produced the same or less TGF-β1 relative to TGF-β2. The significance of these observations is unknown and it is cautionary to note that exceptions were evident (H157, BICR-3, BICR-6).

The autocrine production of the TGF-β isoforms was examined in the context of the genetic background of the cell lines. Cells that overexpressed mutant Ha-*ras* (I-7, II-3, II-4; Boukamp *et al.*, 1990) produced more than twice the amount of TGF-β1 and an absence of TGF-β2 compared with the HaCaT cell line of origin. In I-6 cells, where mutant Ha-*ras* was present but not overexpressed (Boukamp *et al.*, 1990),

TGF-β1 production was increased and TGF-β2 was decreased relative to HaCaT cells. These findings are consistent with the expression of TGF-β1 mRNA in Ha-*ras*-transfected clones of HaCaT cells, but contrast with our previous data where loss of TGF-β protein was noted by II-3 and II-4 cells (Game *et al.*, 1992). In the present study, however, we measured immunogenic TGF-β isoforms rather than total TGF-β concentrated by ultrafiltration (Game *et al.*, 1992). The data obtained for the Ha-*ras*-transfected HaCaT clones in the present study were supported by the findings in the only tumour-derived cell line containing mutant Ha-*ras* where TGF-β1 was markedly enhanced and TGF-β2 undetectable in H357; the level of Ha-*ras* expression in this cell line is currently unknown. Transcriptional activation of the TGF-β1 gene by the Ha-*ras* oncogene has been demonstrated previously (Geiser *et al.*, 1991) but the present report is the first to suggest down-regulation of TGF-β2 expression by mutant Ha-*ras*.

In the present study, the majority of the H series of tumour-derived cell lines were inhibited by TGF-β1, TGF-β2 and TGF-β3. In general, cells that responded to TGF-β1 were also inhibited by TGF-β2, albeit to a lesser extent. The cellular response to TGF-β3 was variable with evidence of inhibition (H357, H376, H400, H413), stimulation (H517) or a lack of response (H103, H314). While such variability may reflect the pattern of expression of cell surface receptors by individual cell lines and/or the cell culture conditions, the results show that a loss of response to one isoform, for example in H314, is invariably associated with a loss of response to the other isoforms of TGF-β. The data also argue against the generally held concept that loss of TGF-β responsiveness is a ubiquitous feature of malignant epithelial cell lines (Fynan and Reiss, 1993).

In general, TGF-β is growth stimulatory for a variety of mesenchymal cells including fibroblasts, chondrocytes and osteoblasts (Roberts and Sporn, 1991). In the present study, fibroblasts from normal oral mucosa were stimulated by TGF-β1, -β2 and -β3, an effect that was more marked after 48 h than 24 h of ligand treatment. By contrast, fibroblasts derived from early cultures of tumour-derived keratinocytes (H357 and H413) lost their stimulatory response to exogenous TGF-β1, -β2 and -β3 such that the cells were less responsive after 48 h compared with 24 h of ligand treatment. Schor *et al.* (1994) have shown that fibroblasts associated with tumour-derived keratinocytes are functionally abnormal, data that support our findings. Interestingly, tumorigenicity of *ras*-transfected primary murine keratinocytes can be suppressed by the addition of normal dermal fibroblasts (Dotto *et al.*, 1988), an effect thought to be mediated by TGF-β3 (Missero *et al.*, 1991). It may be, therefore, that aberrant fibroblasts associated with epithelial tumour development provide a selective growth advantage for tumour cells *in vivo* by failing to produce normal levels of TGF-β3. It was not possible to test this hypothesis because of difficulties in the collection of CM from fibroblasts (loss of cell adhesion and viability occurred in serum-free media) and the lack of sensitivity of the TGF-β3 ELISA. Alternative methods of quantifying TGF-β3 expression, such as RT–PCR, were also explored but were not without difficulty. RT–PCR is at best semi-quantitative and Colletta *et al.* (1990) have shown that TGF-β regulation occurs post-transcriptionally suggesting that protein production should be measured directly rather than by mRNA alone. Nevertheless, we demonstrate TGF-β3 autocrine production by both normal and tumour-derived fibroblast cell lines using RT–PCR. The study of mesenchymal control of epithelial tumour development via TGF-β warrants further investigation in view of the recent work of Shah *et al.* (1995) demonstrating isoform-specific effects of TGF-β in wound healing.

In conclusion, this study demonstrates variable autocrine production of TGF-β1 and -β2 by human tumour-derived and Ha-*ras*-transfected keratinocytes. The peptide was secreted in the latent form. Decreased expression of TGF-β1 was a general characteristic of the tumour-derived cell lines and overexpression of TGF-β1 together with loss of expression of TGF-β2 was a feature of cells containing mutant Ha-*ras*. Keratinocytes were predominantly growth inhibited by TGF-β1, -β2 and -β3, but certain cell lines had a diminished response to the ligands, or were either refractory or stimulated by specific TGF-β isoforms. Further, tumour-associated fibroblasts partially lost a growth-stimulatory response to the TGF-β isoforms. The results demonstrate a marked dysregulation of autocrine and paracrine TGF-β networks in squamous epithelial malignancy.

**Acknowledgements**
The authors wish to thank Professor NE Fusenig for his gift of the Ha-*ras* transfected HaCaT clones. The study was supported by an MRC project grant (G9123775 SD) and Denman's Charitable Trust.

### References

ARTEAGA CL, COFFEY RJ, DUGGER TC, MCCUTCHEN CM, MOSES HL AND LYONS RM. (1990). Growth stimulation of human breast cancer cells with anti-transforming growth factor β antibodies: evidence for negative autocrine regulation by transforming growth factor. *Cell Growth Different.*, **1**, 367–374.

ARTEAGA CL, DUGGER TC, WINNIER AR AND FORBES JT. (1993). Evidence for a positive role of transforming growth factor-β in human breast cancer cell tumorigenesis. *J. Cell. Biochem.*, **17G**, 187–193.

BOUKAMP P., STANBRIDGE EJ, YIN FOO D, CERUTTI PA AND FUSENIG NE. (1990). c-Ha-*ras* oncogene expression in immortalised human keratinocytes (HaCaT) alters growth potential *in vivo* but lacks correlation with malignancy. *Cancer Res.*, **50**, 2840–2847.

BURNS JE, BAIRD MC, CLARK LJ, BURNS PA, EDINGTON K, CHAPMAN C, MITCHELL R, ROBERTSON G, SOUTAR D AND PARKINSON EK. (1993). Gene mutations and increased levels of p53 protein in human squamous cell carcinomas and their cell lines. *Br. J. Cancer*, **67**, 1274–1284.

CHEIFETZ S, HERNANDEZ J, LAIHO M, TEN DIFKE P, IWATA KK AND MASSAGUE J. (1990). Distinct transforming growth factor-β (TGF-β) receptor subsets as determinants of cellular responsiveness to three TGF-β isoforms. *J. Biol. Chem.*, **265**, 20533–20538.

CLARK LJ, EDINGTON K, SWAN IRC, MCLAY KA, NEWLANDS WJ, WILLS LC, YOUNG HA, JOHNSTONE PW, MITCHELL R, ROBERTSON G, SOUTAR D, PARKINSON EK AND BIRNIE GD. (1993). The absence of Harvey *ras* mutations during development and progression of squamous cell carcinomas of the head and neck. *Br. J. Cancer*, **68**, 617–620.

COFFEY RJ, BASCOM CC, SIPES NJ, GRAVES-DEAL R, WEISSMAN BE AND MOSES HL. (1988). Selected inhibition of growth related gene expression in murine keratinocytes by transforming growth factor β. *Mol. Cell Biol.*, **8**, 3088–3093.

COLLETTA AA, WAKEFIELD LM, HOWELL FV, VAN R, DANIELPOUR D, EBBS SR, SPORN MB AND BAUM M. (1990). Anti-oestrogens induce the secretion of active transforming growth factor beta from human fetal fibroblasts. *Br. J. Cancer*, **62**, 405–409.

COOMBS LM, PIGOTT DA, EYDMANN ME, PROCTOR AJ AND KNOWLES MA. (1993). Reduced expression of TGF-β is associated with advanced disease in transitional cell carcinoma. *Br. J. Cancer*, **67**, 578–584.

CIU W, KEMP CJ, DUFFIE E, BALMAIN A AND AKHURST R. (1994). Lack of transforming growth factor-β1 expression in benign skin tumours of $p53^{null}$ mice is prognostic for a high risk of malignant conversion. *Cancer Res.*, **54**, 5831–5836.

DANIELPOUR D. (1993). Improved sandwich enzyme-linked immunosorbent assays for transforming growth factor β1. *J. Immunol. Methods*, **158,** 17–25.

DANIELPOUR D, KIM KY, WINOKUR TS AND SPORN MB. (1991). Differential regulation of the regulation of transforming growth factor-βs 1 and 2 by retinoic acid, epidermal growth factor and dexamethasone in NRK-49F and A549 cells. *J. Cell. Physiol.*, **148,** 235–244.

DERYNCK R, GOEDDEL DV, ULLRICH A, GUTTERMAN JY, WILLIAMS RD AND BRINGMAN TS. (1987). Synthesis of messenger RNAs for transforming growth factor α and β and the epidermal growth factor receptor by human tumours. *Cancer Res.*, **47,** 707–712.

DOTTO GP, WEINBERG RA AND ARIZA A. (1988). Malignant transformation of mouse primary keratinocytes by Harvey sarcoma virus and its modulation by surrounding normal cells. *Proc. Natl Acad. Sci. USA*, **85,** 6389–6393.

EDINGTON KG, LOUGHRAM OP, BERRY IJ AND PARKINSON EK. (1995). Cellular immortality-a late event in the progression of human squamous cell carcinoma of the head and neck associated with p53 alteration and a high frequency of allele loss. *Mol. Carcinogen.*, **13,** 254–265.

FYNAN TM AND REISS M. (1993). Resistance to inhibition of cell growth by transforming growth factor-β and its role in oncogenesis. *Crit. Rev. Oncogen.*, **4,** 493–540.

GAME SM, HUELSON A, PATEL V, DONNELLY M, YEUDALL WA, STONE A, FUSENIG NE AND PRIME SS. (1992). Progressive abrogation of TGF-β1 and EGF growth control is associated with tumour progression in *ras*-transfected human keratinocytes. *Int. J. Cancer*, **52,** 461–470.

GEISER AG, KIM SJ, ROBERTS AB AND SPORN MB. (1991). Characterisation of the mouse transforming growth factor-β1 promoter and activation by the Ha-*ras* oncogene. *Mol. Cell. Biol.*, **11,** 84–92.

GLICK AB, KULKARNI AB, TENNENBAUM T, HENNINGS J, FLANDERS KC, O'REILLY M, SPORN MB, KARLSSON S AND YUSPA SH. (1993). Loss of expression of transforming growth factor β in skin and skin tumours is associated with hyperproliferation and a high risk for malignant conversion. *Proc. Natl Acad. Sci. USA*, **90,** 6076–6080.

GLICK AB, LEE MM, DARWICHE N, KULKARNI AB, KARLSSON S AND YUSPA SH. (1994). Targeted deletion of the TGF-β1 gene causes rapid progression to squamous cell carcinoma. *Genes Dev.*, **8,** 2429–2440.

GORSCH SM, MEMOLI VA, STUKEL TA, GOLD LI AND ARRICK BA. (1992). Immunohistochemical staining for transforming growth factor-β1 associates with disease progression in human breast cancer. *Cancer Res.*, **52,** 6949–6952.

HAFEZ MM, INFANTE D, WINAWER S AND FRIEDMAN E. (1990). Transforming growth factor beta 1 acts as an autocrine-negative growth regulator in colon enterocytic differentiation but not in goblet cell maturation. *Cell Growth Different.*, **1,** 617–626.

JENNINGS JC, MOHAN S, LINKHART TA, WIDSTROM R AND BAYLINK DJ. (1988). Differential activity in endothelial cells. *J. Cell Physiol.*, **137,** 167–172.

KIM SJ, PARK K, KOELLER D, KIM KY, WAKEFIELD LM, SPORN MB AND ROBERTS AB. (1992). Post-transcriptional regulation of the human transforming growth factor-beta 1 gene. *J. Biol. Chem.*, **267,** 13702–13707.

LAWRENCE DA. (1995). Transforming growth factor-β: an overview. *Kidney Int.*, **47,** S19–S23.

LEHMAN TA, MODALI R, BOUKAMP P, STANEK J, BENNETT WP, WELSH JA, METCALF RA, STAMPFER MR, FUSENIG NE, ROGAN EM, REDDEL R AND HARRIS CC. (1993). p53 mutations in human immortalised epithelial cell lines. *Carcinogenesis*, **14,** 833–839.

LEVINE JH, MOSES HL, GOLD LI AND NANNEY LB. (1993). Spatial and temporal patterns of immunoreactive TGF-β1, TGF-β2 and TGF-β3 during excisional wound repair. *Am. J. Pathol.*, **143,** 368–380.

MACCALLUM J, BARTLETT JMS, THOMPSON AM, KEEN JC, DIXON JM AND MILLER WR. (1994). Expression of transforming growth factor-β mRNA isoforms in human breast cancer. *Br. J. Cancer*, **69,** 1006–1009.

MISSERO C, CAJAL SR AND DOTTO GP. (1991). Escape from transforming growth factor β control and oncogene cooperation in skin tumor development. *Proc. Natl Acad. Sci. USA*, **88,** 9613–9617.

MUNGER K, PIETENPOL JA, PITTELKOW MR, HOLT JT AND MOSES HL. (1992). Transforming growth factor β1 regulation of c-*myc* expression, pRB phosphorylation and cell cycle progression in keratinocytes. *Cell Growth Different.*, **3,** 291–298.

PATAMALAI B, BUROW DL, GIMENEZ-CONTI I, ZENKLUSEN JC, CONTI CJ, KLEIN-SZANTO AJP AND FISCHER SM. (1994). Altered expression of transforming growth factor-β1 mRNA and protein in mouse skin carcinogenesis. *Mol. Carcinogen.*, **9,** 220–229.

PELTON RW, SAXENA B, JONES M, MOSES HL AND GOLD LI. (1991). Immunohistochemical localization of TGF-β1, TGF-β2 and TGF-β3 in the mouse embryo: expression patterns suggest multiple roles during embryonic development. *J. Cell Biol.*, **115,** 1091–1105.

PIETENPOL JA, HOLT JT, STEIN RW AND MOSES HL. (1990). Transforming growth factor-β suppression of c-*myc* gene transcription: role in inhibition of keratinocyte proliferation. *Proc. Natl Acad. Sci. USA*, **87,** 3758–3762.

PRIME SS, NIXON SVR, CRANE IJ, STONE A, MATTHEWS JB, MAITLAND NJ, REMNANT L, POWELL SK, GAME SM AND SCULLY C. (1990). The behaviour of human oral squamous cell carcinoma in cell culture. *J. Pathol.*, **160,** 259–269.

PRIME SS, MATTHEWS JB, PATEL V, GAME SM, DONNELLY M, STONE A, PATERSON IC, SANDY JR AND YEUDALL WA. (1994). TGF-β receptor regulation mediates the response to exogenous ligand but is independent of the degree of cellular differentiation in human oral keratinocytes. *Int. J. Cancer*, **56,** 406–412.

ROBERTS AB AND SPORN MB. (1991). The transforming growth factors. In *Peptide Growth Factors and their Receptors I*, Sporn MB and Roberts AB (eds) pp. 419–472. Springer: Berlin.

SCHOR AM, RUSHTON G, FERGUSON JE, HOWELL A, REDFORD J AND SCHOR SL. (1994). Phenotypic heterogeneity in breast fibroblasts: functional anomaly in fibroblasts from histologically normal tissue adjacent to carcinoma. *Int. J. Cancer*, **59,** 25–32.

SHAH M, FOREMAN DM AND FERGUSON MWJ. (1995). Neutralization of TGF-β1 and TGF-β2 or exogenous addition of the TGF-β3 to cutaneous rat wounds reduces scarring. *J. Cell Sci.*, **108,** 985–1002.

SPORN MB AND ROBERTS AB. (1992). Transforming growth factor-B: recent progress and new challenges. *J. Cell. Biol.*, **119,** 1017–1021.

TAKIUCHI H, TADA T, LI X-F, OGATA M, IKEDA T, FUJIMOTO S, FUJIWARA H AND HAMAOKA T. (1992). Particular types of tumour cells have the capacity to convert transforming growth factor β from a latent to an active form. *Cancer Res.*, **52,** 5641–5646.

TEN DIJKE P, IWATA KK, THORIKAY M, SCHWEDES J, STEWART A AND PIELER C. (1990). Molecular characterisation of transforming growth factor type β3. *Ann. NY Acad. Sci.*, **593,** 26–42.

WELCH DR, FABRA A AND NAKAJIMA M. (1990). Transforming growth factor β stimulates mammary adenocarcinoma cell invasion and metastatic potential. *Proc. Natl Acad. Sci. USA*, **87,** 7678–7682.

WU S, THEODORESCU D, KERBEL RS, WILLSON JKV, MULDER KV, HUMPHREY LE AND BRATTAIN MG. (1992). TGF-β1 is an autocrine-negative growth regulator of human colon carcinoma FET cells *in vivo* as revealed by transfection of an anti-sense expression vector. *J. Cell Biol.*, **116,** 187–196.

YEUDALL WA, TORRANCE LK, ELSEGOOD KA, SPEIGHT PA AND PRIME SS. (1993). *Ras* gene point mutation is a rare event in premalignant and malignant lesions of the oral cavity. *Eur. J. Cancer*, **29B,** 63–68.

YEUDALL WA, PATERSON IC, PATEL V AND PRIME SS. (1995). Presence of human papillomavirus sequences in tumour-derived human oral keratinocytes which express mutant p53. *Eur. J. Cancer*, **31B,** 136–143.